# A novel method to derive amniotic fluid stem cells for therapeutic purposes

**DOI:** 10.1186/1471-2121-11-79

**Published:** 2010-10-19

**Authors:** Tatsanee Phermthai, Yuparat Odglun, Suphakde Julavijitphong, Vitaya Titapant, Prakong Chuenwattana, Chanchai Vantanasiri, Kovit Pattanapanyasat

**Affiliations:** 1 Stem Cell Research and Development Unit, Department of Obstetrics & Gynecology, Faculty of Medicine Siriraj Hospital, Mahidol University, Bangkok, 10700, Thailand; 2 Maternal Fetal Medicine Unit, Department of Obstetrics & Gynecology, Faculty of Medicine Siriraj Hospital, Mahidol University, Bangkok, 10700, Thailand; 3 Office for Research and Development, Faculty of Medicine Siriraj Hospital, Mahidol University, Bangkok, 10700, Thailand

## Abstract

**Background:**

Human amniotic fluid stem (hAFS) cells have become an attractive stem cell source for medical therapy due to both their ability to propagate as stem cells and the lack of ethical debate that comes with the use of embryonic stem cells. Although techniques to derive stem cells from amniotic fluid are available, the techniques have limitations for clinical uses, including a requirement of long periods of time for stem cell production, population heterogeneity and xeno-contamination from using animal antibody-coated magnetic beads. Herein we describe a novel isolation method that fits for hAFS derivation for cell-based therapy.

**Methods and Results:**

With our method, single hAFS cells generate colonies in a primary culture of amniotic fluid cells. Individual hAFS colonies are then expanded by subculturing in order to make a clonal hAFS cell line. This method allows derivation of a substantial amount of a pure stem cell population within a short period of time. Indeed, 10^8 ^cells from a clonal hAFS line can be derived in two weeks using our method, while previous techniques require two months. The resultant hAFS cells show a 2-5 times greater proliferative ability than with previous techniques and a population doubling time of 0.8 days. The hAFS cells exhibit typical hAFS cell characteristics including the ability to differentiate into adipogenic-, osteogenic- and neurogenic lineages, expression of specific stem cell markers including Oct4, SSEA4, CD29, CD44, CD73, CD90, CD105 and CD133, and maintenance of a normal karyotype over long culture periods.

**Conclusions:**

We have created a novel hAFS cell derivation method that can produce a vast amount of high quality stem cells within a short period of time. Our technique makes possibility for providing autogenic fetal stem cells and allogeneic cells for future cell-based therapy.

## Background

With the hope of using stem cells for medical therapy, research and understanding of many aspects of stem cell biology has increased extensively. Stem cells from many sources have been explored for their advantages and limitations in clinical use. There are significant limitations in the use of adult tissue stem cells and embryonic stem cells. Specifically, for adult tissue stem cells, only a small amount of stem cells are able to be obtained, and these cannot be effectively propagated. The use of embryonic stem cells (ESC) is hindered by ethical concerns, feeder cell requirements and teratoma formation. Thus, a new source of human stem cells for use in clinical purposes is needed.

Amniotic fluid (AF) cells are the heterogeneous cell population of exfoliated fetal and amniotic cells [1], which are routinely harvested by amniocentesis for fetal genetic determination in prenatal diagnosis. In 2003, Prusa *et al. *[2] reported the discovery of OCT-4 positive cells in amniotic fluid, which is a pluri-potent characteristics. The biology of human amniotic fluid stem (hAFS) cells was subsequently explored in several reports [1,3-6]. The potency of hAFS cells seems to be between pluripotent ESC and adult stem cells, the cells express some pluri-potent stem cell markers. The hAFS cells can grow in a simple culture without a feeder cell requirement. They have high *in vitro *proliferation potential (over 250 population doublings with a doubling time of 1.6 days). Moreover, hAFS cells are not subject to teratocarcinoma formation and ethical debates [1,2,5]. These characteristics make hAFS cells an attractive source for providing a variety of major histocompatibility complex immunity. Their broad spectrum ability of lineage differentiation and specialized function has been reported in all three germ layers [3,5]. Thus, AF is an appropriate source of stem cells for clinical purposes.

The first technique to derive hAFS cells was developed in 2004 by Tsai *et al. *[1], who reported a two-stage culture technique. With the protocol, non-adherent cells from routine amniocentesis were used for hAFS cell derivation, but the yield showed heterogeneity within the hAFS cell population. In 2006, Tsai *et al. *[3] established an optional protocol following the two-stage culture method for generating high population purity by constructing a clonal hAFS cell line from a single hAFS cell. Subsequently, Kim *et al. *(2007) [4] presented a protocol for deriving hAFS cells. The technique is performed by prolonging an *in vitro *hAFS cell culture with subsequent subculturing until a stem cell population with a homogeneous morphology can be obtained. In 2007, De Coppi *et al. *[5] demonstrated a hAFS cell isolation protocol based on the principle of immunoselection. This method specifically selected the c-Kit positive stem cells from amniotic fluid using magnetic cell sorting and was followed by clonal cell culture. This immunoselection technique is efficient for producing a high purity hAFS cell population, but the process utilizes xeno-antibodies and micromagnetic beads.

Although several hAFS cell derivation techniques have now been developed, the existing techniques are unsuitable for hAFS production for medical purposes because these methods often result in contamination with other cell types or contamination with antibodies raised from animals. Additionally, these techniques require a long period of time for stem cell production. Hence, a better method which allows utilization of these cells for cell-based therapy needs to be developed.

In the current study, we present the starter cell method as an efficient technique that is suitable to derive hAFS cells for therapeutic purposes.

## Methods

### Derivation of hAFS cells with the starter cell method

Five milliliters of fifteen independent amniotic fluid samples were obtained from 16-20 week pregnant women who underwent amniocentesis for fetal genetic determination in routine prenatal diagnosis. The study protocol was approved by the Ethics Committee of Siriraj hospital, Mahidol University, Thailand and each participant received an informed consent document. Cells were immediately isolated from amniotic fluid and cell debris by centrifugation at 2,100 rpm for 5 min. The pellet cells were resuspended with 5 ml of Chang medium (Irvine Scientific, CA, USA), which has been used in routine amniocentesis culture. A resuspension was plated on a 100 mm^2 ^tissue culture dish (Nunc, NY) and incubated at 37°C in humidified 5% CO_2_, 5% O_2 _for 3-4 days. To identify the starter cells, the distinct fibroblast-like cells in the primary culture dish were observed under inverted microscope. The individual single hAFS cells were cultured continuously as starter cells to form hAFS cell colonies. To keep each colony at the proper distance, only one starter cell was allowed to exist under a microscopic field using 10× magnifications. Unneeded starter cells were mechanical removed by pipette tip. For a colony forming from a starter cell, only the starter cell was continued in culture. The medium and non-adherent cells were removed from the primary culture dish. Five milliliters of hAFS cell medium containing α-MEM medium (Gibco, Invitrogen, CA) supplemented with 15% ES-FBS (PAA, Pasching, Austria), 1% glutamine (Sigma, MO), 1% penicillin/streptomycin (Biochrom, Berlin, Germany), 18% Chang B and 2% Chang C (Irvine Scientific, CA) and 10 ng/ml bFGF (Chemicon, Millipore, MA) was added to the culture dish. The culture was maintained in the primary culture dish for 48 h. The cells in each colony were counted in order to predict and select a clone for hAFS cell expansion. Next, each hAFS cell colony was mechanically picked up under inverted microscope using fine-tipped pipettes. The cells in each hAFS cell colony were re-seeded into a well of a 24-well plate. The culture was performed at 37°C under humidified 5% CO_2_, 5% O_2_. When a clonal hAFS reached confluence at 70% of the culturing area, subculturing was performed by trypsinization and re-plating into a 25 cm^2 ^tissue culture flask. The medium was changed every other day. The hAFS cells were allowed to expand to 70% confluence and then routinely subcultured with a dilution of 1:3.

### Immunofluorescent staining of hAFS cells

hAFS cell markers were characterized using fluorescence microscope detection and flow cytometry analysis. For immunofluorescence, hAFS cells at 70% confluence were fixed with 4% paraformaldehyde (Merck, Darmstadt, Germany). Cells were stained overnight with primary antibody against Oct-4a (Santa Cruz Biotechnology, CA) and SSEA-4 (Chemicon, Millipore, MA). The cells were then washed twice with PBS-Tween (PBS+0.5% Tween-20; UBS, OH) before staining with a 1:200 dilution of rabbit anti-human IgG secondary antibody (Chemicon, Millipore, MA). The specificity of each reaction was visualized by inverted fluorescent microscopy. For flow cytometry analysis, hAFS cells were harvested and stained with FITC or PE-conjugated antibodies against CD29, CD44, CD90, CD105, CD133 (E-bioscience, CA), CD34, CD45, CD73, SSEA-4 (Beckton Dickinson, NJ) and Oct-4a. The cells were fixed with 1% paraformaldehyde. The analysis was performed using a Beckton Dickinson flow cytometer (Beckton Dickinson, NJ).

### Reverse-transciptase PCR

Total RNA was extracted from hAFS cells by Phenol-Chloroform and used as a template for reverse transcription. The cDNA was made by using RevertAid First Strand cDNA Synthesis Kit (Fermentas, EU). The primers used in PCR are as follows:

Oct-4 (247 bp) sense, 5'-CGTGAAGCTGGAGAAGGAGAAGCTG-3', and antisense, 5'-CAAGGGCCGCAGCTTACACATGTTC-3';

HLA-ABC (394 bp) sense, 5'-GTATTTCTTCACATCCGTGTCCCG-3', and antisense, 5'-GTCCGCCGCGGTCCAAGAGCGCAG-3';

HLA-DR (220 bp) sense, 5'-CTGATGAGCGCTCAGGAATCATGG-3', and antisense, 5'-GACTTACTTCAGTTTGTGGTGAGGGAAG-3';

Nestin (395 bp) sense, 5'-CCAGAAACTCAAGCACCAC-3', and antisense, 5'-TTTTCCACTCCAGCCATCC-3';

Nanog (161 bp) sense, 5'-AGTCCCAAAGGCAAACAACCCACTTC-3', and antisense, 5'-

TGCTGGAGGCTGAGGTATTTCTGTCTC-3';

Sox2 (449 bp) sense, 5'-CCCCCGGCGGCAATAGCA-3', and antisense, 5'-TCGGCGCCGGGAGATACAT-3';

and beta-Actin (107 bp) sense, 5'-ATGTGGCCGAGGACTTTGATT-3', and antisense, 5'-AGTGGGGTGGCTTTTAGGATG-3'.

The cDNA amplification for Oct-4 and HLA-ABC were performed by the following PCR conditions: initial denaturing at 95°C for 5 min and 35 cycles of 94°C for 45 sec, 57°C for 1 min, 70°C for 1 min and extension at 70°C for 10 min. For HLA-DR, Nestin, Nanog, Sox2 and β-actin, the PCR conditions were as follows: 95°C for 5 min and 35 cycles of 94°C for 45 sec, 52°C for 1 min, 70°C for 1 min and extension at 70°C for 10 min.

### Differentiation

To investigate the differentiation capacity, hAFS cells were *in vitro *differentiated into three different cell specific lineages, including adipogenic, osteogenic and neurogenic lineages. This hAFS cells were cultured in hAFS medium until 70% confluence and then shifted to a specific induction medium under the same condition of 5% CO_2_, 5% O_2 _at 37°C. The medium was changed twice a week. For differentiation to osteogenic and adipogenic lineages, the obtained cells were cultured in the appropriate medium. The osteogenic medium contains alpha-MEM (Gibco) supplemented with 10% ES-FBS (PAA), 0.1 μM dexamethasone (Sigma), 10 mM glycerol-2-phosphate (Sigma) and 50 μM ascorbic acid (Sigma). The adipogenic medium contains alpha-MEM (Gibco) supplemented with 10% ES-FBS (PAA), 1 μM dexamethasone (Sigma), 5 μg/ml insulin (Sigma), 0.5 mM 3-isobutyl-1-methylxanthine (Sigma), and 60 μM indomethacin (Sigma). For neural differentiation, hAFS cells were cultured in neurogenic inducing medium containing alpha-MEM (Gibco) supplemented with 20% ES-FBS (PAA), 1 μM beta-mercaptoethanol (Sigma), and 5 ng/ml basic FGF (Invitrogen) for 24 h and then shifted to serum-depleted medium (alpha-MEM, 10 uM beta-mercaptoethanol) for 5 h.

### Immunochemical staining of differentiated cells

The osteogenesis was assessed by alkaline phosphatase enzyme activity. Calcium accumulation was examined using von Kossa staining. The Oil Red O staining was used for detection of intracellular lipid droplet formation for evaluating adipogenesis. For evaluation of neural differentiation, the neuron specific class III β-tubulin (TuJ-1) was used.

## Results

**Figure 1:**
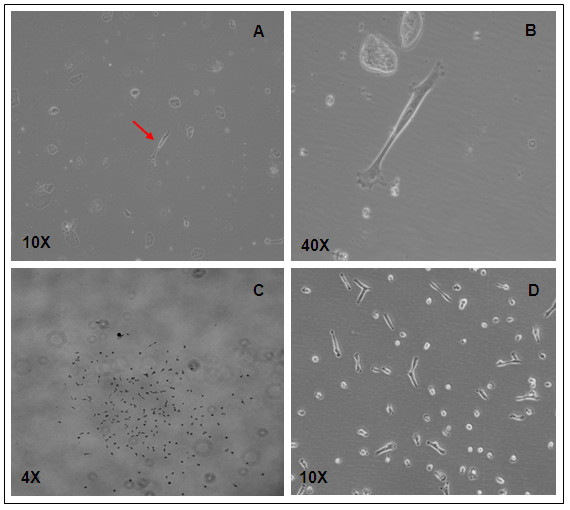
**Derivation of clonal hAFS cell lines by the starter cell method**. (A) and (B) A hAFS starter cell of fibroblastic type (arrow) was found early after a routine amniocentesis culture, (C) the colony appearance of a clonal hAFS cell line at 48 h in the primary culture dish, (D) morphology of hAFS cells derived by the starter cell method at subculture passage 3.

Almost all samples (14 out of 15) showed success for derivation of clonal hAFS cell lines. At day 3 of primary culture of the independent hAF samples, we found four to eight single hAFS cells adhering in a distinct area of the culture dish. A hAFS cell in fibroblastic cell type is shown in Figures 1A and 1B. Each single cell was used for hAFS cell colony expansion, which we denoted as the "starter cell". After allowing growth in hAFS medium for 48 h, each formed a colony. Each colony contained about 100-300 cells (Figure 1B). The morphologic characteristics of the hAFS cell colonies were as follows: 1) each cell within a colony stayed equidistant from other cells within that same colony, 2) most cells in a colony were in metaphase, and 3) cells in colonies showed the morphology of fibroblast-like cells (Figure 1C). An individual colony in primary culture was expanded as a clonal hAFS cell line. Each independent colony was removed and re-seeded into a 24-well culture plate as the first subculture passage (passage 1). The clonal hAFS reached 70% confluence (about 30,000-35,000 cells) by 3-4 days after cell seeding. The hAFS cells can be expanded up to 10^7 ^cells within a following week by subsequent culturing to passage 2 in a 25 cm^2 ^culture flask and then 75 cm^2 ^culture flasks. Using our method, 10^10 ^cells from a clonal hAFS cell population can be derived within weeks.

**Figure 2:**
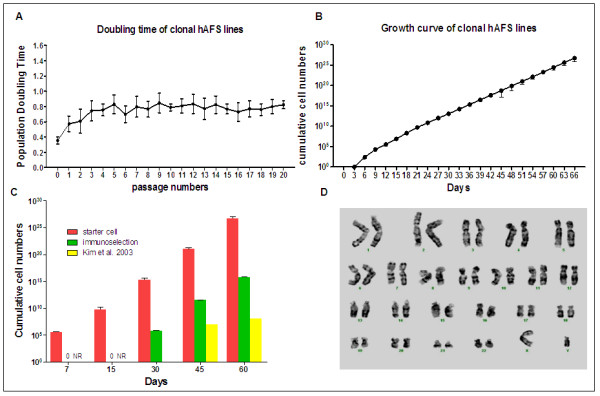
**Proliferative ability curves and chromosomal stability**. (A) The population doubling time pattern of a clonal AFS cell line established by the starter cell method was investigated from primary culture at passage 0 to passage 20. The greatest proliferative ability of hAFS cells was found at passage 0 and subsequently declined after trypsinization. (B) The average cumulative cell number of ten hAFS cell lines derived by the starter cell method at the different days is shown in a growth curve. (C) The cumulative hAFS cell numbers derived from three methods were compared. The green block is the cumulative number for the hAFS cell line, which was derived by the protocol described in [5]. The data for the yellow block was used from the report by Kim *et al. *in [4]. NR means no data reported. (D) A clonal AFS cell line at passage 18 was investigated for chromosomal stability after prolonged *in vitro *culture. The normal karyotype of a clonal AFS cell line is shown by Giemsa staining.

Notably, we found that the resultant hAFS cells have the highest proliferation potential in primary culture (passage 0), with a population doubling time of 0.3 days. The proliferative ability was gradually reduced during early subculture passages (from passage 0 to passage 4). The population doubling time at passage 1, 2 and 3 were 0.5, 0.6 and 0.8 days, respectively. After passage 4, hAFS cells showed a stable rate of cell proliferation with a doubling time of about 0.8 days. A normal karyotype of 46XX and 46XY was observed in clonal hAFS cells at passage 18. The growth rate, population accumulation and population doubling time of the resultant hAFS cells are shown in Figures 2A and 2B. The hAFS cells derived by our technique and two other previous techniques were compared based on their accumulation numbers, and this data is presented in Figure 2C.

The selected starter cell is a fibroblast-like cell, which was obtained from an adherent stem cell on day 3 of the primary human amniotic fluid cell culture. We did often find contamination with other cell types in the human AF samples, such as epithelial cells, which can form colonies. However, the morphological appearance of these colonies is obviously different from the hAFS cell colonies. Over 85% of the adherent single cells in the primary culture dish at day 3 are hAFS cells, with fibroblastic morphology. Eighty to one hundred percent of single hAFS cells can be *in vitro *extended and form a good quality clonal hAFS line.

**Figure 3:**
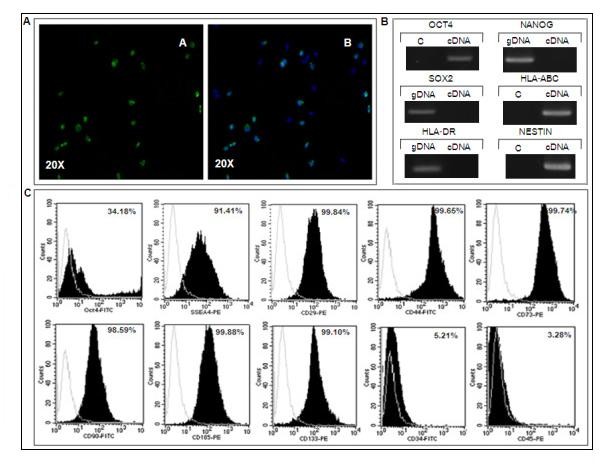
**Characteristics of hAFS cells obtained by the starter cell method**. (A) Clonal hAFS cells at passage 18 show an Oct-4 specific signal after staining with FITC-conjugated secondary antibody against anti-Oct4a with fluorescent microscope at 20× magnification. The Oct-4 positive signal is exhibit in hAFS population as shown by merge of Oct-4a and Hoechst 33342 staining. (B) The hAFS cell line at passage 18 has expression of Oct-4a and HLA-ABC, but not HLA-DR, Nanog and Sox2 by cDNA analysis using RT-PCR. The expression of Nestin was observed in hAFS cell-derived neurons. C means negative control, while gDNA was used as positive control of PCR amplification. (C) The flow cytometry analysis shows expression of SSEA4, CD29, CD44, CD73, CD90, CD105 and CD133, but not CD34 or CD45 on the hAFS cell surface.

The hAFS cells were evaluated for pluripotent characteristic by expression of the Oct-4a transcription factor by immunocytochemical examination and RT-PCR. The clonal hAFS cell lines at passage 18 were used for these studies. Immunofluorescence detection showed that cells in a clonal population had a positive signal for Oct-4a (Figure 3A). Gene expression analysis revealed a PCR product for Oct-4 with a size of 247 bp, but not Nanog and Sox2 (Figure 3B). In addition, we analyzed hAFS cells for expression of immunological factors, and analysis yielded a PCR product for HLA-ABC with a product size of 394 bp using RT-PCR; however, we did not observe expression of HLA-DR (Figure 3B). More antigenic characteristics of hAFS cells were investigated by flow cytometry analysis. The results demonstrated high positive signals for mesenchymal stem cell markers CD29, CD44, CD73, CD105, CD133 but low signals for CD90. The hAFS cells show a negative signal for hematopoietic stem cell markers CD34 and CD45 on the surface of hAFS cells (Figure 3C). For detection of pluri-potent embryonic stem cell markers using flow cytometer, 24-41% of cells within the hAFS cell population expressed OCT-4a, whereas 48-91% of the cell population had a positive SSEA-4 signal. Homogeneity of the obtained hAFS cell population was observed by flow cytometry, in which 99-100% of cells within the population expressed CD29, CD44, CD73 and CD105.

**Figure 4:**
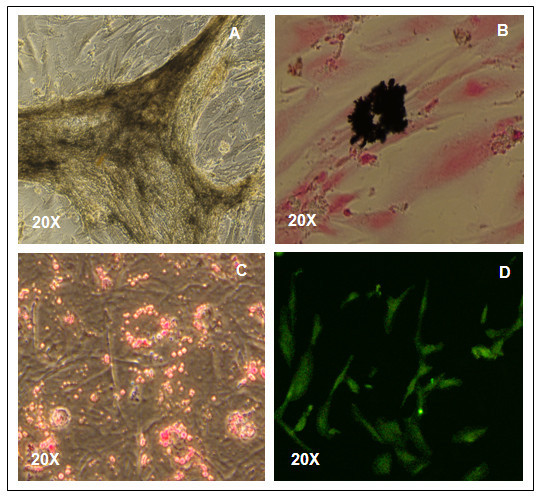
**Staining of hAFS cell-derived multi-lineage cells**. A clonal hAFS cell line at passage 12 derived by the starter cell method was *in vitro *induced with proper induction mediums and shows the appearance of lineage-specific signals of osteoblasts by staining with Alkaline Phosphatase (A) and Von Kossa (B). The lineage-specific signals of adipocytes and neurons exhibit after staining with Oil Red O (C) and Tuj-1 (D), respectively. 20× magnification was used.

To evaluate the differentiation ability of clonal hAFS cells derived by our method, the cells were *in vitro *induced into adipocytes, osteoblasts and neurons by lineage-specific induction mediums. For osteogenic differentiation, the cells showed positive alkaline phosphatase staining after incubation in osteogenic induction medium for 3 weeks (Figure 4A). The calcium mineralization of hAFS-derived osteogenic cells was verified by a photochemical reaction using von Kossa staining (Figure 4B). For adipogenic differentiation, the clonal hAFS cells showed morphologic changes at day 6 under adipogenic induction medium. The appearance of endogenous lipid droplets was observed by staining with Oil Red O after 2 weeks of culture (Figure 4C). For neurogenic differentiation, the cells appeared to have the morphology of neural-like cells after incubating with neurogenic inducing medium and serum-depletion medium for 7 days. Finally, the cells showed positive staining for neuron-specific class III β-tubulin (Figure 4C). The expression of Nestin was found in with RT-PCR product sizes of 395 bp (Figure 3B).

## Discussion

We have developed an efficient method to derive hAFS cells for medical therapy. The requirements for stem cells for use in clinical therapy include a pure stem cell population, good cell quality, no animal product contamination and rapid clonal hAFS cell production. Our derivation technique was designed to meet these requirements. The technique starts by selecting adherent stem cells from a primary hAF culture. A selective individual hAFS cells is called a "starter cell". The starter cell is used as a beginner cell for generating a clonal hAFS cell line. In previous techniques, hAFS cell line establishment utilizes primary hAF cell cultures, hAF stem cell isolation and deriving a clonal line from a single hAFS cell. With the starter cell method, these three steps of AFS derivation protocol can be combined into a single step. This makes the protocol simpler and shorter. In addition, the obtained clonal AFS cell line has high homogeneity. Thus, the starter cell method creates high opportunity for providing hAFS cells that fit for medical therapy.

For selection of the starter cells in a hAF primary culture, we utilized hAF cells which were actively proliferating. Based on the principles of cell division as described in [7], dividing cells have increased cell mass due to duplication of organelles, macromolecules and DNA, and synthesis of the mitosis proteins for chromosome doubling and cell dividing in the G2 and M phases. The coordination of cell division renders the release of cell membrane stiffness, which leads to the attachment of floating exfoliated AF cells on a culture dish in the early days of culture.

The immunoselection method demonstrated in De Coppi *et al. *[5] is an efficient technique by which to derive clonal hAFS cell lines from a heterogeneous cell population. The hAFS cells derived by this technique are suitable only for research study due to the use of antibodies raised from animals. The antibodies contaminate the stem cell population, as they are of foreign origin. In order to make cells that are safe for human use, our method was performed without xeno-immunologic substances, allowing further technical developments for a xeno-free production system.

The primary cells found in amniotic fluid are a heterogeneous population due to their varied origins. The varied origins of the cells result in different degrees of cell differentiation depending on the stage of organ development. Only 1% of cells in amniotic fluid demonstrate stem cell characteristics. In previous methods, the derivation protocol begins with routine amniocentesis and allowing the co-culturing of heterogeneous hAF cells. We suspect that this step of amniocentesis culture may unsuitable for hAFS cell derivation. The co-culturing of heterogeneous hAF cells is similar to induction of differentiation using conditioned medium. The conditioned medium is provided by the culturing of primary cells and then enriched with lineage-specific growth factors. The conditioned medium can induce stem cell differentiation via intracellular signaling pathways. Several differentiation studies have shown good results by using this induction approach [8-11]. From this, we understand that co-culturing of heterogeneous hAF cells may affect the quality of the stem cells.

In the hAFS cell derivation method modified by Kim *et al. *[4], the hAFS cells are co-cultured with AF primary cells for a long period of time. The population doubling time of hAFS cells derived by this protocol was shown to be 3.6 days. In contrast, the technique developed by De Coppi *et al. *[5] cultures stem cells with a heterogeneous population of primary AF cells for a short period of time before immunoselection. These hAFS cells showed a doubling time of 1.6 days.

Nevertheless, the starter cell method was designed to avoid the culture of stem cells with a heterogeneous population in primary culture. The obtained clonal hAFS cell lines have a high proliferative ability and a 0.8 day population doubling time. This is 2-5 times faster than the population doubling time with previous techniques reported by De Coppi *et al. *[5] and Kim *et al. *[4]. The hAFS cells derived from our method show a steady pattern of cell doubling over 20 subculture passages with maintenance of a normal karyotype. They also maintain the ability to differentiate and have stem cell marker expression, including Oct-4, SSEA-4, CD29, CD44, CD73, CD90, CD105, and CD133. These data suggest a superior quality of the hAFS cells derived by our method.

Each independent amniotic fluid sample contributes a number of clonal hAFS cell lines using previous isolation techniques. However, assessments of hAFS cell lines have not been performed. Stem cell selection was performed by random choosing of colonies, which often results in inefficient cell lines. The selective line can also be derived by repeated culture, which requires a higher investment in money and time. Derivation of clonal hAFS cell lines using the starter cell method can help to eliminate the problem of random selection and the previously mentioned waste according to predict cell line quality from outgrowth colony of each starter cell in primary culture. Furthermore, our method is a simple protocol that does not require a chemical kit, thus making it more cost effective.

The hAFS cells found in our study are consistent with those identified by Tsai *et al. *(2004) [1] and Kim *et al. *(2007) [4], both of whom suggested that amniotic fluid stem cells are morphologically a fibroblast cell type.

## Conclusions

We report the starter cell method as a simple approach to provide good quality hAFS cells that fit the requirements for use in therapeutic purposes. This technique should allow the generation of fetal autogenic cell and allogeneic support for future cell-based therapy. The further step required for development of hAFS to clinical level is elimination of xeno-component in hAFS cells culture condition. Providing of xeno-free hAFS cells should make the possibility and safety of hAFS cells for medical therapy.

## Competing interests

The authors declare that they have no competing interests.

## Authors' contributions

TP contributed the concept and design of the protocol, stem cell isolation, culture and differentiation, fluorescence microscope studies, collection and assembly of data, data analysis and interpretation as well as writing and making the manuscript. YO carried out stem cell culture and differentiation as well as fluorescence microscope studies and participated in the flow cytometry analysis. SJ carried out stem cell differentiation, staining studies and helped manuscript. VT carried out sample collection and participated in AFS cell isolation and culture by the immunoselection technique. PC carried out sample collection and participated in AFS cell isolation and culture by immunoselection technique. CV carried out the chromosomal and molecular genetic analyses. KP carried out the flow cytometry analysis. All authors read and approved the final manuscript.

## References

[B1] Tsai MS Lee JL Chang YJ Hwang SM Isolation of human multipotent mesenchymal stem cells from second-trimester amniotic fluid using a novel two-stage culture protocol Hum Reprod 2004 19 1450 1456 10.1093/humrep/deh279 15105397

[B2] Prusa AR Marton E Rosner M Bernaschek G Hengstschläger M Oct-4-expressing cells in human amniotic fluid: a new source for stem cell research? Hum Reprod 2003 18 1489 1493 10.1093/humrep/deg279 12832377

[B3] Tsai MS Hwang SM Tsai YL Cheng FC Lee JL Chang YJ Clonal amniotic fluid-derived stem cells express characteristics of both mesenchymal and neural stem cells Biol Reprod 2006 74 545 551 10.1095/biolreprod.105.046029 16306422

[B4] Kim J Lee Y Kim H Hwang KJ Kwon HC Kim SK Cho DJ Kang SG You J Human amniotic fluid-derived stem cells have characteristics of multipotent stem cells Cell Prolif 2007 40 75 90 10.1111/j.1365-2184.2007.00414.x 17227297 PMC6496664

[B5] De Coppi P Bartsch G Jr Siddiqui MM Xu T Santos CC Perin L Mostoslavsky G Serre AC Snyder EY Yoo JJ Furth ME Soker S Atala A Isolation of amniotic stem cell lines with potential for therapy Nat Biotechnol 2007 25 100 106 10.1038/nbt1274 17206138

[B6] You Q Cai L Zheng J Tong X Zhang D Zhang Y Isolation of human mesenchymal stem cells from third-trimester amniotic fluid Int J Gynaecol Obstet 2008 103 149 152 10.1016/j.ijgo.2008.06.012 18760782

[B7] Alberts B Johnson A Lewis J Raff M Roberts K Walter P The cell cycle and programmed cell death Molecular biology of the cell 2002 4 New York: Garland Science, a member of the Taylor & Francis group 985 1026

[B8] Kitazawa A Shimizu N Differentiation of mouse embryonic stem cells into neurons using conditioned medium of dorsal root ganglia J Biosci Bioeng 2005 100 94 99 10.1263/jbb.100.94 16233857

[B9] Zhang JQ Yu XB Ma BF Yu WH Zhang AX Huang G Mao FF Zhang XM Wang ZC Li SN Lahn BT Xiang AP Neural differentiation of embryonic stem cells induced by conditioned medium from neural stem cell Neuroreport 2006 17 981 986 10.1097/01.wnr.0000227977.60271.ca 16791088

[B10] Lacham-Kaplan O Chy H Trouson A Testicular cell conditioned medium supports differentiation of embryonic stem cells into ovarian structures containing oocytes Stem cells 2006 24 266 273 10.1634/stemcells.2005-0204 16109761

[B11] Bordoni V Alonzi T Zanetta L Khouri D Conti A Corazzari M Bertolini F Antoniotti P Pisani G Tognoli F Dejana E Tripodi M Hepatocyte-conditioned medium sustains endothelial differentiation of human hematopoietic-endothelial progenitors Hepatology 2007 45 1218 1228 10.1002/hep.21568 17464995

